# Heterogeneous pathways to depressive and anxiety disorders: A cluster-based predictive study in a nationwide longitudinal cohort

**DOI:** 10.1017/S0033291726104590

**Published:** 2026-05-14

**Authors:** Chong Chen, Yoshiyuki Asai, Yasuhiro Mochizuki, Kosuke Hagiwara, Ryo Okubo, Shin Nakagawa, Takahiro Tabuchi

**Affiliations:** 1 Division of Neuropsychiatry, Department of Neuroscience, Yamaguchi University Graduate School of Medicine, Ube, Yamaguchi, Japan; 2 Department of Systems Bioinformatics, Yamaguchi University Graduate School of Medicine, Ube, Japan; 3 Center for Data Science, Waseda University, Shinjuku-ku, Tokyo, Japan; 4 Department of Psychiatry, Hokkaido University Graduate School of Medicine, Sapporo, Hokkaido, Japan; 5 Division of Epidemiology, School of Public Health, Graduate School of Medicine, Tohoku University, Sendai, Miyagi, Japan

**Keywords:** anxiety, data-driven, depression, hierarchical clustering, machine learning, mental health, random forest, risk factor, unsupervised learning

## Abstract

**Background:**

Early prediction of depressive and anxiety disorders is challenging due to substantial heterogeneity in risk pathways. Conventional machine-learning models trained on aggregated populations may obscure subgroup-specific mechanisms and limit interpretability for prevention. We evaluated whether a hybrid unsupervised–supervised framework can identify meaningful subgroups and yield more interpretable risk prediction.

**Methods:**

We analyzed cohort data of 15,897 Japanese adults who completed baseline (August–September 2020) and 6-month follow-up (February–March 2021) surveys and did not screen positive for depressive and anxiety disorders at baseline (K6 score < 13). Using 169 baseline demographic, psychosocial, lifestyle, and behavioral variables, we performed hierarchical clustering to derive data-driven subgroups. Within each cluster, we trained Random Forest models to predict incident screened depressive and anxiety disorders at follow-up (K6 ≥ 13) and interpreted predictors using SHapley Additive exPlanations (SHAP).

**Results:**

The overall 6-month incidence was 6.23%. A five-cluster solution revealed two high-risk subgroups: an older-adult profile with poor quality of life (12.9%) and a working-parent profile characterized by work–family overload (29.8%). Compared with a global model trained on the full sample, the cluster-then-predict framework showed broadly similar overall performance but performed better in the highest-risk subgroup and revealed more differentiated predictor profiles. Loneliness, health-related quality of life, happiness, and personality traits predominated in clusters with moderate adversity, whereas lifestyle disruption (sleep, diet, and irregular routines) characterized the high-risk late-life subgroup and alcohol dependence and work–family burden characterized the high-risk working-parent subgroup.

**Conclusions:**

Addressing risk-factor heterogeneity before prediction may enable more interpretable, context-tailored prevention strategies.

## Introduction

Depressive and anxiety disorders are among the most common mental health conditions and remain leading causes of functional impairment worldwide (World Health Organization, 2017). Beyond subjective distress, they are associated with reduced workplace productivity and substantial societal costs (Chen et al., 2024; Evans-Lacko & Knapp, 2016; World Health Organization, 2022). During periods of child-rearing, they may also disrupt family functioning and adversely affect child wellbeing, which causes longer-term intergenerational consequences (Callender, Olson, Choe, & Sameroff, 2012; Hirai et al., 2025; Lawrence, Murayama, & Creswell, 2019). These broad impacts emphasize the importance of identifying vulnerability before symptoms become clinically entrenched.

Prevention research further indicates that interventions are most effective when targeted to individuals with heightened vulnerability or early signs of distress rather than applied uniformly across the population (Cuijpers et al., 2021). This creates a strong rationale for developing scalable approaches to identify elevated risk before substantial functional decline.

In recent years, machine learning has emerged as a promising tool for predicting the onset of depressive and anxiety disorders using demographic, behavioral, lifestyle, and psychosocial information (Chen & Nakagawa, 2025; Li et al., 2024; Na, Cho, Geem, & Kim, 2020; Song et al., 2023). Such models can capture nonlinear associations and higher-order interactions that traditional approaches may miss. However, despite methodological advances, prediction performance and interpretability have often remained insufficient for clinical or policy implementation. A major reason is the substantial heterogeneity underlying depressive and anxiety disorders (Hollon, Andrews, & Thomson, 2021; Lynall & McIntosh, 2023; Nandi, Beard, & Galea, 2009; Spokas & Cardaciotto, 2014). Individuals may reach similar symptom thresholds through different combinations of social adversity, health behavior patterns, occupational stress, family burden, and psychological dispositions. When models are trained on an aggregated population, subgroup-specific mechanisms may be averaged out, which dilutes risk signals and makes results harder to translate into actionable prevention strategies (Dwyer, Falkai, & Koutsouleris, 2018).

Addressing heterogeneity is therefore critical. One promising solution is to identify meaningful subgroups before prediction so that risk models are learned within more homogeneous contexts (Dwyer et al., 2018). Unsupervised clustering methods can uncover latent groupings in high-dimensional data without imposing predefined strata such as age or employment categories (Hastie, Tibshirani, & Friedman, 2009). If such subgroups reflect coherent life-stage and psychosocial contexts, then subgroup-specific supervised models may yield clearer, more interpretable determinants of vulnerability. This approach may support prevention strategies that are better aligned with people’s lived circumstances.

In this study, we propose a hybrid unsupervised–supervised machine-learning framework to address heterogeneity in the risk of depressive and anxiety disorders in a nationwide longitudinal cohort of Japanese adults. We first apply hierarchical clustering to a broad set of baseline psychosocial, lifestyle, and behavioral measures to derive data-driven subgroups. We then train and interpret cluster-specific supervised models to predict incident depressive and anxiety disorders 6 months later. To evaluate the added value of this framework, we also compare its predictive performance and SHAP-based feature importance profiles with those of a single global supervised model trained on the full sample. By integrating population stratification with subgroup-specific prediction, we aim to reveal context-specific pathways to vulnerability and provide a foundation for subgroup-tailored prevention strategies.

## Methods

### Participants

We used data from the Japan COVID-19 and Society Internet Survey (JACSIS), a nationwide online survey conducted in August–September 2020 (Okubo et al., 2021), with follow-up in February–March 2021 as part of the Japan Society and New Tobacco Internet Survey (JASTIS; follow-up rate: 81.57%; Tabuchi et al., 2019). Participants were recruited from a large web-based panel using random sampling stratified by sex, age, and prefecture. After quality control and exclusions (Supplementary methods), 17,141 adults aged 20 to 79 years remained. Of these, 15,897 participants did not screen positive for depressive or anxiety disorders at baseline (Kessler Psychological Distress Scale [K6] score below 13; Kessler et al., 2003; Furukawa et al., 2008) and were included in the current analyses. Notably, the sample distribution by sex, age, and prefecture was highly comparable to the Japanese population (Supplementary Figure S1). Participants provided web-based written informed consent, and the study protocol was approved by the Research Ethics Committee of the Osaka International Cancer Institute.

### Outcomes and predictors

**Figure 1.**
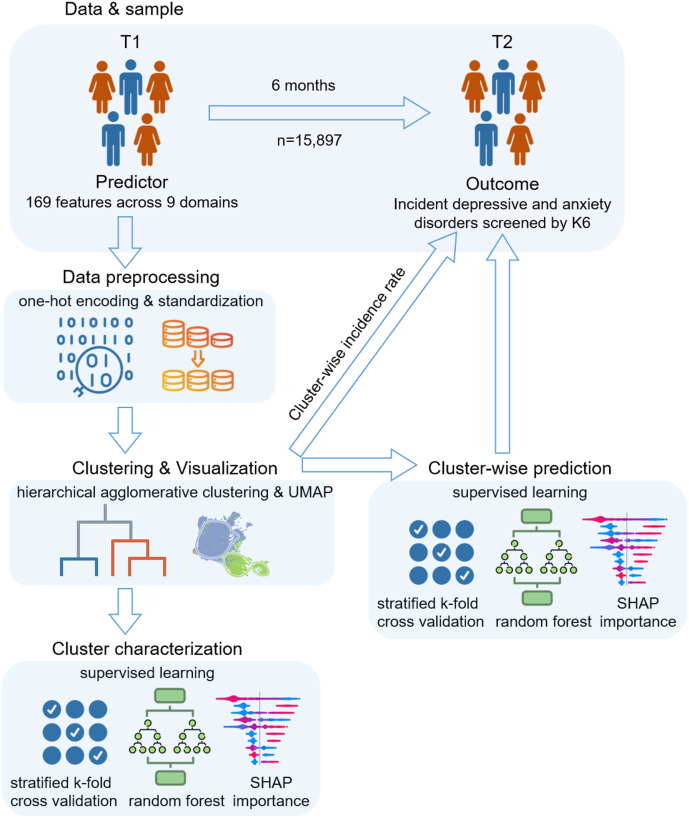
Analytical workflow of the study. Participants who did not screen positive for depressive or anxiety disorders (K6 < 13) at baseline were analyzed using 169 predictors. After preprocessing and standardization, hierarchical clustering (Ward) was performed, and the optimal k was selected based on composite internal metrics. UMAP visualizations illustrate subgroup structure. Cluster characteristics were then examined using Random Forests and SHAP values, and separate predictive models were trained within each cluster to evaluate risk factors for incident disorders. Icons from Flaticon.

The analytical workflow is shown in Figure 1. The outcome was incident-screened depressive and anxiety disorders at follow-up, which was operationalized as scoring 13 or higher on the Kessler Psychological Distress Scale (K6; Kessler et al., 2003; Furukawa et al., 2008). The Japanese adaptation of the K6 has demonstrated excellent diagnostic performance and achieved an area under the receiver operating characteristic curve (ROC AUC) of 0.94 (95% CI: 0.88–0.99) in distinguishing DSM-IV (American Psychiatric Association, 1994) depressive (including major depressive disorder and dysthymia) and anxiety disorders (including panic disorder, agoraphobia, social phobia, generalized anxiety disorder, and post-traumatic stress disorder; Furukawa et al., 2008).

Predictors comprised 169 baseline measures covering demographic, health, psychological factors, finances, family, lifestyle, work, social factors, and COVID-19–related variables (details provided in Supplementary Table S1).

### Data preprocessing

Continuous, ordinal, and binary variables were used as recorded. Non-binary nominal variables were one-hot encoded. Psychometric scales were entered as total or subscale scores. For items not applicable to certain respondents (e.g. marital questions for individuals who are single), we set these responses to zero and added a corresponding indicator for non-applicability (although none of these indicators appeared among the top predictors; see Figure 4; Supplementary Figure S2). Duplicated variables and features with identical values were removed. For clustering and Uniform Manifold Approximation and Projection (UMAP) visualization, numeric variables were standardized.

### Clustering and visualization

We performed hierarchical agglomerative clustering using Ward’s minimum variance criterion (Murtagh & Legendre, 2011; Ward, 1963) on the preprocessed baseline feature matrix. Candidate solutions (k = 2–20) were evaluated using four widely used internal validation indices: within-cluster sum of squared errors (Lloyd, 1982), Silhouette coefficient (Rousseeuw, 1987), Calinski–Harabasz index (Caliński & Harabasz, 1974), and Davies–Bouldin index (Davies & Bouldin, 1979). Indices were normalized and averaged to form a composite score, and the k maximizing this score was selected. After the cluster solution was selected, we examined cluster-wise follow-up incidence of screened depressive and anxiety disorders as a post hoc descriptive external characterization of the derived clusters. Because our primary goal was to capture risk factor heterogeneity, we expected the emergence of clusters with differential psychological vulnerability. Cluster structure was visualized post hoc using two- and three-dimensional UMAP (McInnes, Healy, & Melville, 2018), only for illustrative purposes.

### Cluster characterization using supervised learning

To quantify the separability of clusters and identify distinguishing features, we trained a Random Forest classifier (Breiman, 2001) to predict cluster membership from baseline predictors. Hyperparameters were tuned using Optuna’s Tree-structured Parzen Estimator (Akiba et al., 2019) to maximize stratified five-fold cross-validated balanced accuracy. Specifically, the dataset was randomly divided into five approximately equal folds while preserving the distribution of cluster labels across folds. In each iteration, four folds were used for model training and hyperparameter tuning, and the remaining fold was used as the held-out validation fold. This procedure was repeated five times so that each fold served once as the validation fold, and model performance was summarized based on the cross-validated results. Feature contributions were interpreted using SHapley Additive exPlanations (SHAP) with TreeExplainer (Lundberg et al., 2020), which quantifies the marginal contribution of each feature to the prediction of cluster membership.

### Cluster-wise prediction of incident disorders using supervised learning

Within each cluster, we trained separate Random Forest models to predict incident depressive and anxiety disorders at follow-up. To account for the relatively low outcome prevalence, we used *class_weight =* ‘*balanced*’ so that the minority class received greater weight during model training. Models were tuned with Optuna to maximize ROC AUC using stratified cross-validation (number of folds adapted to cluster event counts). Using out-of-fold predicted probabilities, we computed ROC AUC and PR AUC and selected an optimal decision threshold via Youden’s J index (Youden, 1950). We then reported clinically relevant classification metrics, including sensitivity, specificity, precision, negative predictive value, F1 score, and balanced accuracy. For all performance metrics, 95% bootstrap confidence intervals were estimated from 2,000 bootstrap resamples of the out-of-fold predictions; for threshold-based metrics, the cluster-specific threshold derived from the original out-of-fold predictions was held fixed during bootstrapping. For interpretation, SHAP values for the outcome class were computed within each cluster, and key predictors were compared across clusters using a heatmap of mean absolute SHAP values.

### Benchmark comparison with a global model

To benchmark the proposed cluster-then-predict framework, we additionally trained a single global Random Forest model on the full sample using the same predictors, preprocessing steps, class weighting, hyperparameter tuning procedure, and cross-validation framework as in the cluster-specific analyses. Model performance was also evaluated in the same way. To compare the global and cluster-specific approaches, we first compared the global model with the combined out-of-fold predictions from the cluster-specific models across all participants, with each participant assigned the prediction from the model corresponding to their own cluster. We then evaluated the global model within each cluster and compared its cluster-wise performance with that of the corresponding cluster-specific model. To compare interpretability, we additionally computed SHAP values for the global model and contrasted its feature importance profile with those of the cluster-specific models.

Analyses were conducted in Python (details in Supplementary Methods).

## Results

### Unsupervised clustering and cluster characterization

**Figure 2.**
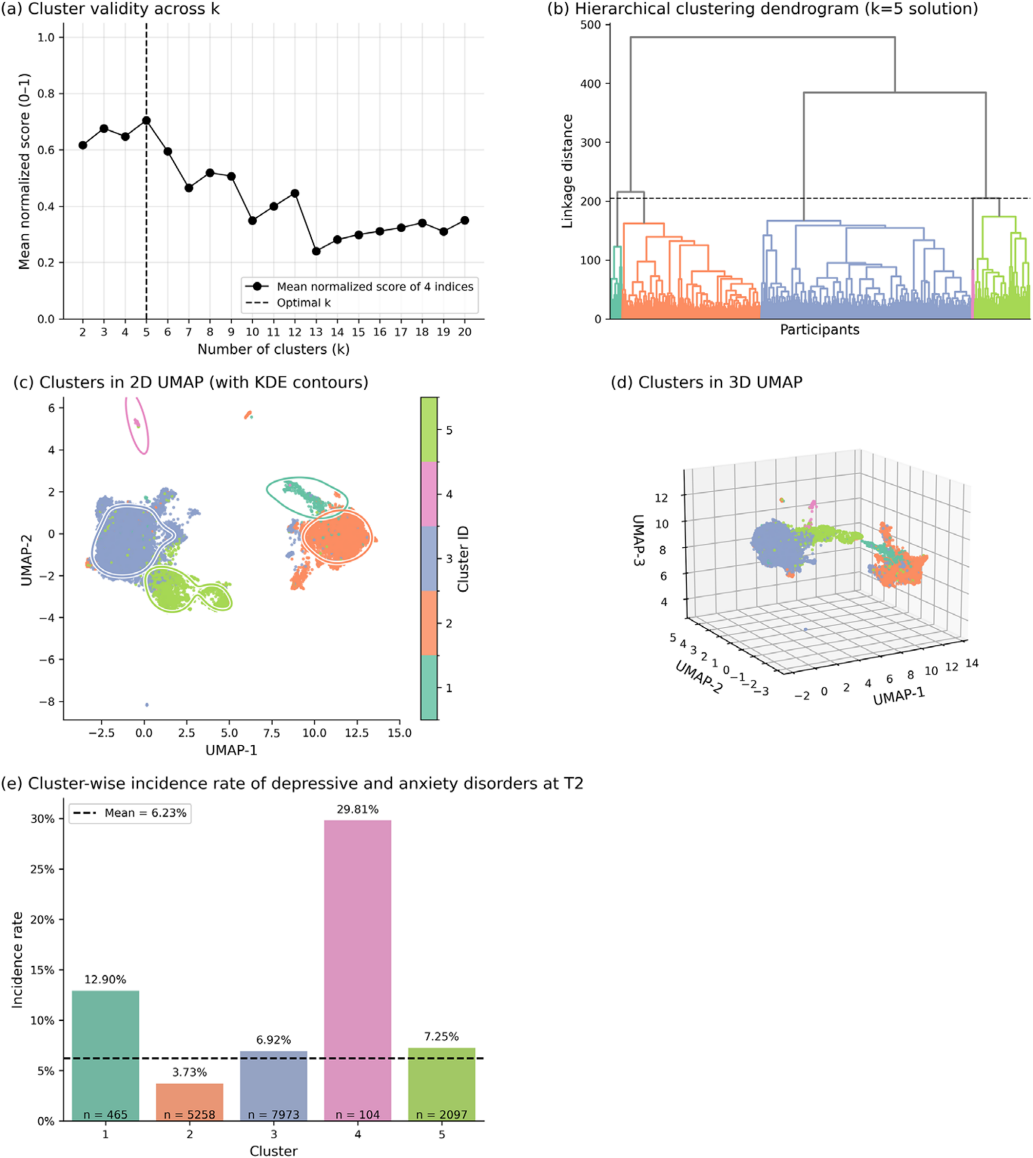
Overview of cluster validity, structure, and the incidence of depressive and anxiety disorders across subgroups. (a) Mean normalized cluster validity score (composite of within-cluster sum of squares, Silhouette, Calinski–Harabasz, and Davies–Bouldin indices) across candidate numbers of clusters (k = 2–20). The vertical dashed line indicates the optimal solution (k = 5). Consistent with the composite criterion, the Davies–Bouldin index also independently favored k = 5. (b) Hierarchical clustering dendrogram based on Ward’s method, with the horizontal dashed line indicating the cut level corresponding to the five-cluster solution. (c) Two-dimensional Uniform Manifold Approximation and Projection (UMAP) embedding colored by cluster ID, with kernel density contours outlining high-density regions within each cluster. (d) Three-dimensional UMAP embedding of the same clusters. (e) Cluster-wise incidence rates of screened depressive and anxiety disorders at follow-up (T2), with bars colored by cluster, sample sizes displayed at the base of each bar, and percentages shown above. The horizontal dashed line indicates the mean incidence across all participants.

Internal validation indices supported a five-cluster solution (Figure 2a,b), which also showed clear separation in two- and three-dimensional UMAP embeddings (Figure 2c–d). Incidence of depressive and anxiety disorders at follow-up differed substantially across clusters (Figure 2e). Whereas the overall incidence was 6.23% in the whole sample, two clusters showed markedly elevated risk: Cluster 1 (*n* = 465) 12.90% and Cluster 4 (*n* = 104) 29.81%. Cluster 2 (*n* = 5,258) had the lowest incidence (3.73%), while Clusters 3 and 5 (*n* = 7,973 and 2,097) were near the sample mean (6.92% and 7.25%, respectively).

**Figure 3.**
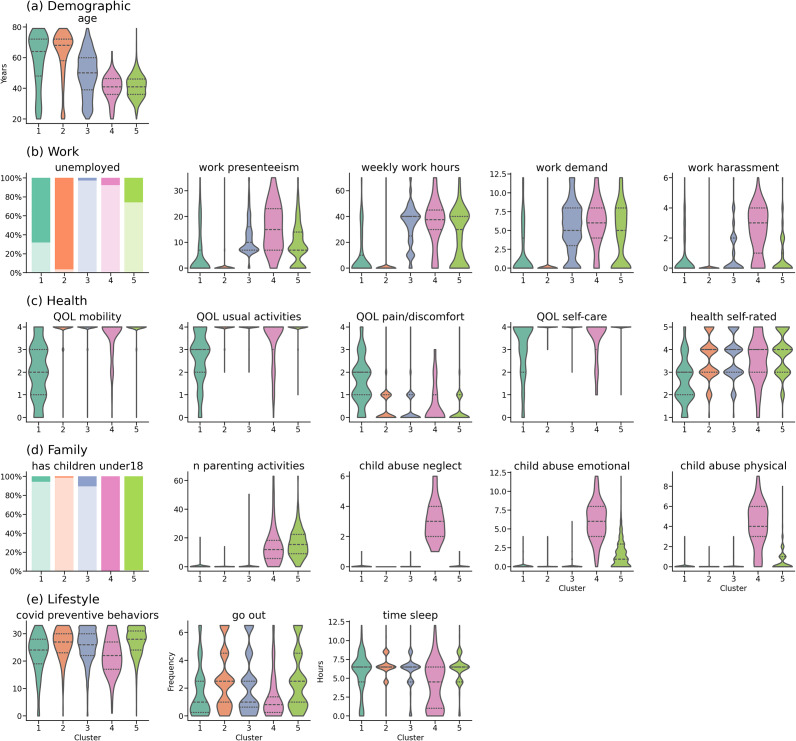
Distribution of SHAP-identified differentiating features across clusters. The 40 most globally important features (ranked by mean absolute SHAP values) were grouped into five domains: demographic, work, health, family, and lifestyle. To improve readability, only key differentiating features are shown for the work and family domains. For continuous and ordinal variables, violin plots show the distribution of feature values by cluster, including median and interquartile ranges. For binary and one-hot-encoded variables, stacked bar charts show the proportion of participants with values 0 (translucent segment) and 1 (solid segment) in each cluster, with bar color indicating cluster ID. Distributions of all 40 features in the original SHAP rank order are provided in Supplementary Figure S3.

Cluster membership was predictable from baseline features (Random Forest balanced accuracy ≥0.898 across clusters; Supplementary Figure S2), which supports that the clusters are operationally characterizable. To summarize these cluster profiles, we grouped the most differentiating baseline features (identified by SHAP) into five domains (demographic, work, health, family, and lifestyle) (Figure 3; full distributions in Supplementary Figure S3). Cluster 1 (*late-life adversity*) included adults mostly in their 50s–70s, often unemployed (~70%), with poor health and quality of life and lower engagement in COVID-19 preventive behaviors. Cluster 2 (*late-life low risk*) was also dominated by older adults in their 60s to 70s and largely unemployed, but otherwise lacked strong distinguishing features in other domains. Cluster 3 (*working non-parents*) encompassed individuals of all age groups and was characterized by high employment. Cluster 4 (*work–family overload*) consisted of working parents in their 30s–40s with high job strain (e.g. presenteeism, work demands, workplace harassment) and marked family stress (including child-abuse behaviors), along with lifestyle disruptions (e.g. lower engagement in preventive behaviors, fewer outings, and shorter sleep). Cluster 5 (*working parents, low strain*) included parents of similar age but with low occupational and family strain.

### Cluster-wise prediction of incident depressive and anxiety disorders using supervised learning

**Figure 4.**
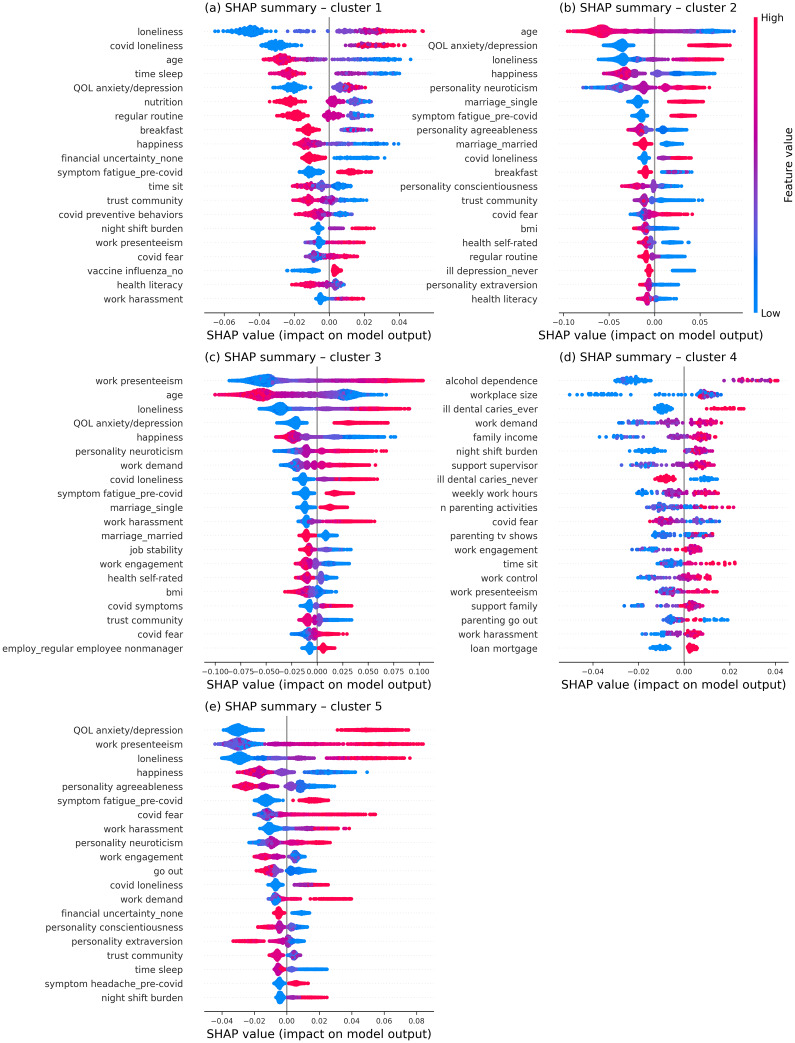
Cluster-specific SHAP feature importance for predicting incident depressive and anxiety disorders at follow-up. Beeswarm plots showing SHAP (SHapley Additive exPlanations) value distributions for the top 20 features contributing to the Random Forest models within each cluster. Each point represents an individual participant, and its horizontal position indicates the feature’s marginal contribution to higher (positive SHAP) or lower (negative SHAP) predicted risk within that cluster. Color indicates feature values (high in red, low in blue). Features are ranked vertically by their overall impact, with those at the top contributing most strongly. Panels (a)–(e) correspond to Clusters 1–5.

We trained cluster-specific Random Forest models to predict incident disorders at follow-up. Performance was consistently robust across clusters (ROC AUC ≥0.788; balanced accuracy ≥0.733; full metrics in Supplementary Figure S4). Confidence intervals were wider for smaller clusters (i.e. Clusters 1 and 4), which is consistent with the limited sample size and the smaller absolute number of positive cases in those clusters. SHAP analyses indicated both shared and cluster-specific predictors (Figure 4). Across Clusters 1–3 and 5, risk was primarily driven by younger age, poorer quality of life, and loneliness, whereas Cluster 4 (*work–family overload*) showed a distinct pattern dominated by alcohol dependence and work- and parenting-related stressors.

**Figure 5.**
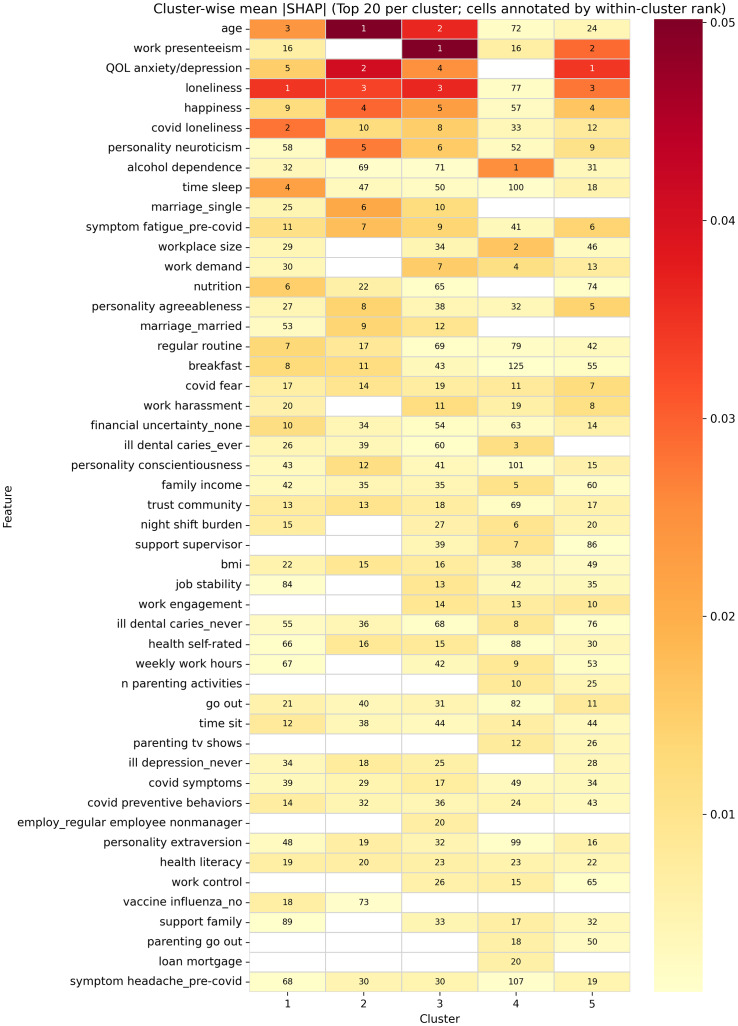
Cross-cluster comparison of mean absolute SHAP importance for predicting the incidence of depressive and anxiety disorders at follow-up. Heatmap displays the union of the top 20 features from each cluster-specific Random Forest model, with rows ordered by the maximum mean |SHAP| observed across clusters. Columns correspond to clusters, and cell color indicates the mean absolute SHAP value for that feature within that cluster (warmer colors = greater importance). White cells indicate near-zero importance (|mean SHAP| below 0.001). Numbers within colored cells denote the within-cluster rank of that feature’s mean |SHAP| (1 = most important) in the corresponding cluster.

Cross-cluster comparison (Figure 5) showed limited overlap in the top predictors: fear of COVID-19 was the only feature consistently appearing among the top 20 predictors across all clusters. Several features were repeatedly important to Clusters 1–3 and 5, including QOL (anxiety/depression domains), loneliness, happiness, COVID-related loneliness, pre-pandemic fatigue, and trust in the local community. Personality traits of neuroticism, agreeableness, conscientiousness, and extraversion contributed strongly to Clusters 2, 3, and 5 but not to the two highest-risk clusters (Clusters 1 and 4).

Cluster-specific predictors further highlighted divergent risk pathways. Cluster 1 (*late-life adversity*) was characterized by a predominance of lifestyle-related predictors, including shorter sleep, poorer nutrition, irregular routines, unhealthy breakfast habits, fewer COVID-preventive behaviors, and lower health literacy. Cluster 4 (*work–family overload*) was predicted by alcohol dependence and work–family burden, including larger workplace size, higher job demand, night-shift burden, longer weekly working hours, and intensive parenting activities. Cluster 4 also had other distinctive predictors, such as a history of dental caries and mortgage loans, suggesting additional psychosocial and health–behavior risks. Clusters 2 (*late-life low risk*) and 3 (*working non-parents*) additionally reflected socioeconomic vulnerability, such as being single, lower BMI, and poorer self-rated health. However, each also had unique predictors: Cluster 2 was more strongly associated with personality traits (e.g. agreeableness and conscientiousness), whereas Cluster 3 was characterized by job instability, non-managerial employment status, and COVID-related symptoms. Cluster 5 (*working parents, low strain*) showed contributions from shorter sleep, financial uncertainty, reduced frequency of going out, and more frequent pre-pandemic headaches.

### Comparison with a global model

To benchmark the proposed framework, we trained a global Random Forest model on the full sample using the same modeling pipeline as in the cluster-specific analyses. At the overall level, predictive performance was broadly similar and slightly favored the global model (ROC AUC = 0.871, 95% CI [0.861, 0.880]; balanced accuracy = 0.796, 95% CI [0.785, 0.808]) over the cluster-then-predict framework (ROC AUC = 0.855, 95% CI [0.845, 0.864]; balanced accuracy = 0.781, 95% CI [0.769, 0.793]) (full metrics in Supplementary Figure S5). Thus, stratifying the sample before prediction did not yield a marked overall improvement in discrimination across the entire cohort.

At the cluster level, however, differences emerged (Supplementary Figure S5). For Clusters 1, 2, 3, and 5, the performance of the global and cluster-specific models was generally similar, with only modest differences across metrics. In contrast, in Cluster 4 (work–family overload), which had the highest follow-up incidence of depressive and anxiety disorders (29.81%), the cluster-specific model showed a clearer advantage. Specifically, it achieved higher ROC AUC (0.795 [0.696, 0.880] versus 0.643 [0.523, 0.759]), PR AUC (0.629 [0.461, 0.788] versus 0.437 [0.293, 0.615]), specificity (0.918 [0.847, 0.973] versus 0.534 [0.423, 0.648]), precision (0.739 [0.550, 0.909] versus 0.414 [0.286, 0.542]), F1 score (0.630 [0.462, 0.767] versus 0.539 [0.400, 0.660]), and balanced accuracy (0.733 [0.637, 0.823] versus 0.654 [0.555, 0.750]) than the global model, although recall was lower (0.548 [0.370, 0.724] versus 0.774 [0.609, 0.923]). These findings suggest that a cluster-specific approach may be particularly useful in subgroups with more distinctive and concentrated risk structures.

SHAP comparisons further identified differences in interpretability (Supplementary Figures S6 and S7). The top features in the global model largely overlapped with those of the larger clusters, particularly Clusters 2, 3, and 5. In contrast, several predictors that ranked highly in Cluster 4, and to a lesser extent Cluster 1, were much lower-ranked or nearly absent in the global model. For example, Cluster 4-specific predictors such as alcohol dependence and several work- and family-related burden indicators were strongly weighted in the cluster-specific model but were attenuated in the global model. Similarly, some lifestyle-related predictors that were prominent in Cluster 1 showed weaker contributions in the global model. Together, these results indicate that the global model mainly captured dominant predictor patterns from the larger subgroups, whereas the cluster-specific models more clearly represented subgroup-specific risk profiles.

## Discussion

In this large longitudinal cohort of Japanese adults, we found that a hybrid unsupervised–supervised machine-learning framework can meaningfully clarify heterogeneity in the risk of depressive and anxiety disorders. By first partitioning individuals into data-driven subgroups and then applying cluster-specific predictive modeling, we achieved high interpretability. Five distinct clusters emerged, each reflecting coherent life-stage, psychosocial, and contextual patterns. These subgroups differed markedly in their 6-month incidence of depressive and anxiety disorders, with two high-risk clusters showing a two- to five-fold elevation in risk and one cluster demonstrating reduced vulnerability. Together, these results underscore the benefits of stratifying heterogeneous populations prior to predictive modeling and suggest that risk for common mental disorders is frequently expressed through subgroup-specific pathways that may be obscured when individuals are analyzed as a single aggregated population.

The five-cluster solution revealed interpretable and non-arbitrary contextual profiles. The *late-life adversity* cluster comprised older adults with reduced physical and psychological quality of life and lower engagement in preventive health behaviors, whereas the *work–family overload* cluster represented working parents in their 30s–40s exposed to intense occupational strain, parenting burden, and lifestyle disruption. These two clusters exhibited the highest incidence of depressive and anxiety disorders, indicating two distinct high-risk trajectories: late-life functional vulnerability and cross-domain work*–*family overload. These patterns are consistent with evidence linking late-life functional decline to depression (Blazer & Hybels, 2005; Bruce, 2002; Haigh, Bogucki, Sigmon, & Blazer, 2018) and excessive role strain among working parents to deteriorating mental health (Allen, Herst, Bruck, & Sutton, 2000; Yucel & Borgmann, 2022). Importantly, these profiles emerged organically from unsupervised analysis of multi-domain life circumstances, without imposing any predefined strata such as age or employment status.

In contrast, the *late-life low-risk* cluster and *working parents, low-strain* cluster shared demographic features with the high-risk clusters but exhibited attenuated stressor intensity and substantially lower risk. This contrast highlights that vulnerability is driven not by demographic identity per se, but by the accumulation and interaction of contextual stressors. The *working non-parents* cluster, covering all age groups, was characterized by balanced life circumstances and showed average risk, which further highlights the contextual nature of vulnerability.

Cluster-specific predictions further demonstrated that determinants of risk differed meaningfully by subgroup. In clusters with moderate adversity – *late-life low risk*, *working non-parents*, and *working parents, low strain* – psychosocial indicators, particularly loneliness, health-related quality of life, and personality traits, were dominant predictors. Neuroticism, conscientiousness, and extraversion contributed strongly to risk prediction in clusters with moderate adversity but were largely absent from the two highest-risk clusters (*late-life adversity* and *work–family overload*), suggesting that trait-based vulnerability is most salient when contextual stressors remain tolerable, whereas structural stressors dominate when adversity becomes severe or multidimensional. From a theoretical perspective, this pattern is broadly consistent with diathesis–stress models (Monroe & Simons, 1991), which emphasize the joint contribution of individual susceptibility and environmental stressors, and with context–trait interaction accounts (Spokas & Cardaciotto, 2014), which suggest that dispositional vulnerabilities may be expressed differently across contexts or subgroups. However, our results also extends these frameworks by indicating that the predictive salience of personality traits may diminish when adversity becomes severe or multidimensional.

In contrast, the *work–family overload* cluster showed a distinct profile in which alcohol dependence, and occupational and parenting burden were prominent, along with additional psychosocial and health-behavior risks such as history of dental caries and mortgage loans. This constellation of predictors is consistent with a stress accumulation mechanism, whereby sustained cross-domain overload erodes psychological resilience (Allen et al., 2000; Yucel & Borgmann, 2022). The *late-life adversity* cluster showed a different high-risk pattern dominated by lifestyle-related factors such as shorter sleep, poorer diet, and irregular routines, suggesting that elevated vulnerability can arise through multiple context-dependent routes (Hollon et al., 2021).

Notably, overlap in the top predictors across clusters was limited, which supports the idea that risk depends on socioecological context and that subgroup-specific mechanisms may be obscured when models are trained on aggregated populations (Dwyer et al., 2018).

These findings have practical implications for prevention. A ‘one-size-fits-all’ approach to screening and intervention may miss subgroup-specific leverage points, whereas selective and indicated strategies can be better aligned with individuals’ risk contexts (Cuijpers et al., 2021). For instance, older adults with functional and lifestyle vulnerabilities may benefit from strategies targeting sleep, daily routines, health literacy, and social connection. Working parents with high work–family strain may require interventions that extend beyond individual-level support to organizational and structural changes, including workload management, harassment prevention, and childcare-related supports (Allen et al., 2000; Yucel & Borgmann, 2022).

Our hybrid approach advances a methodological argument: heterogeneity may be informative to address before prediction, even when doing so does not uniformly improve overall predictive performance. In our benchmark comparison, the cluster-then-predict framework showed broadly similar overall performance to a single global model trained on the full sample, with the global model showing slightly better overall discrimination. However, the cluster-specific approach yielded a notable performance advantage in Cluster 4, the highest-risk subgroup, and produced more differentiated predictor profiles across subgroups. Traditional machine-learning models are generally trained on entire populations, which implicitly average across divergent subgroups and may unintentionally dilute risk signals (Dwyer et al., 2018). In contrast, stratifying the population first may be valuable because it can reveal subgroup-specific pathways to risk and improve performance in selected high-risk subgroups. This approach is well aligned with precision psychiatry goals (Bzdok & Meyer-Lindenberg, 2018; Salagre & Vieta, 2021).

Precision was relatively low for most clusters, which should be interpreted in light of both the relatively low incidence of the outcome and our choice of tuning metric. Precision is sensitive to class imbalance, and when the positive class is infrequent, even models with reasonable discriminative ability may yield modest precision. In addition, our hyperparameter tuning was based on ROC AUC rather than PR AUC, and thus did not directly optimize precision. We selected ROC AUC as the primary tuning metric because it is a widely used and recommended measure of discrimination in medical prediction research and captures performance across the full sensitivity–specificity tradeoff (Hajian-Tilaki, 2013; Van Calster et al., 2025). In screening contexts such as ours, this tradeoff is clinically relevant because missed true cases may have important consequences, although ROC AUC should still be interpreted alongside other performance measures rather than in isolation.

Several limitations should be considered. First, all measures were self-reported, which may introduce reporting bias for sensitive domains (e.g. alcohol problems or abusive behaviors). Second, the outcome was based on the K6 screening threshold, an established proxy for depressive and anxiety disorders (Furukawa et al., 2008; Kessler et al., 2003), rather than a clinical diagnosis. Replication using diagnostic interviews is needed. Third, baseline assessments occurred during the early phase of the COVID-19 pandemic, and the subgroup structure and predictors may partly reflect that context (Brooks et al., 2020; Craig & Churchill, 2021; Santini et al., 2020). Fourth, cluster solutions may depend on the available features and cultural setting (Dwyer et al., 2018), and external validation in other cohorts is essential. Finally, the current work evaluates a 6-month horizon, and longer-term stability of subgroup-specific prediction requires further study.

## Conclusion

In this nationwide longitudinal cohort, an integrated unsupervised–supervised framework identified data-driven subgroups with distinct risk profiles for depressive and anxiety disorders. Cluster-specific modeling revealed context-dependent predictors, suggesting multiple pathways to vulnerability and supporting more targeted prevention strategies than one-size-fits-all approaches. Replication and longer-term validation are needed to translate these subgroup-based models into practice.

## Supporting information

10.1017/S0033291726104590.sm001 Chen et al. supplementary material Chen et al. supplementary material

## Supplementary material

The supplementary material for this article can be found at http://doi.org/10.1017/S0033291726104590.
