# Supplementary material for: Heterogeneous pathways to depressive and anxiety disorders: A cluster-based predictive study in a nationwide longitudinal cohort
Source: Psychol Med. 2026 May 14;56:e139. doi: 10.1017/S0033291726104590 (PMC13177010; doi:10.1017/S0033291726104590)
Supplement: Chen et al. supplementary material [file S0033291726104590sup001.docx]

Risk Factor Heterogeneity in Depression and Anxiety Disorders Using Cluster-Based Prediction

Supplementary material

**Figure S1**. Comparison of the demographic distribution of the analyzed sample with that of the Japanese population aged 20–79 years

**Table S1**. Key predictor variables used in the current study

**Figure S2**. SHAP-based characterization of cluster-defining features and cluster classification performance

**Figure S3**. Distributions of SHAP-identified top 40 globally important features across clusters

**Figure S4**. Cluster-wise performance of Random Forest models predicting incident depressive and anxiety disorders at follow-up

**Figure S5.** Comparison of predictive performance between the global model and the cluster-then-predict framework

**Figure S6.** SHAP feature importance for the global Random Forest model predicting incident depressive and anxiety disorders at follow-up

**Figure S7.** Comparison of SHAP feature importance between the global model and cluster-specific models for predicting incident depressive and anxiety disorders at follow-up

**Supplementary methods**


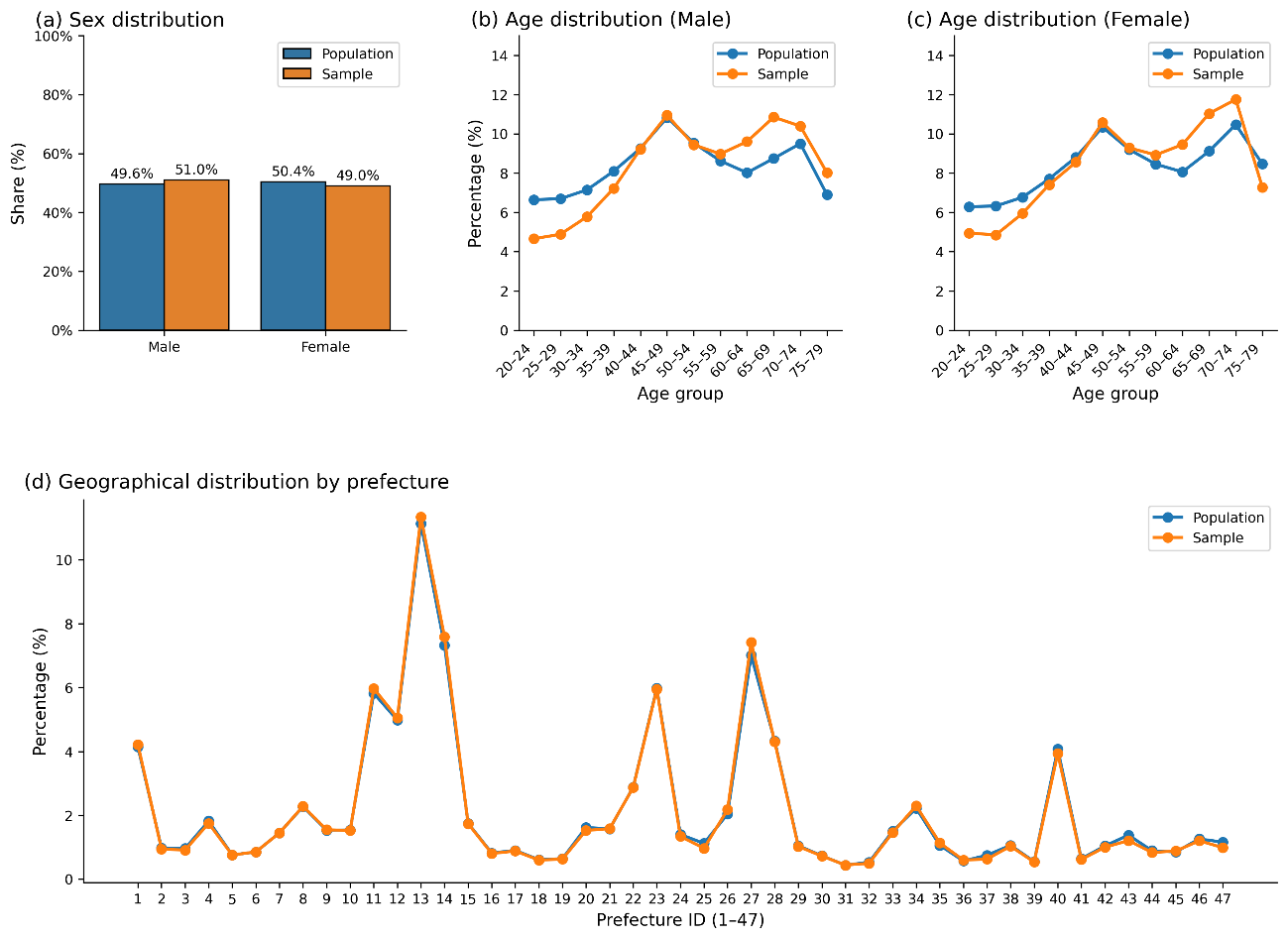


**Figure S1. Comparison of the demographic distribution of the analyzed sample with that of the Japanese population aged 20–79 years.** (a) Sex distribution, (b) age distribution for males, (c) age distribution for females, and (d) prefecture-level geographical distribution. Blue lines represent the distribution of the Japanese adult population, and orange lines represent the distribution of the present sample. The 47 Japanese prefectures are numbered as follows: 1 Hokkaido, 2 Aomori, 3 Iwate, 4 Miyagi, 5 Akita, 6 Yamagata, 7 Fukushima, 8 Ibaraki, 9 Tochigi, 10 Gunma, 11 Saitama, 12 Chiba, 13 Tokyo, 14 Kanagawa, 15 Niigata, 16 Toyama, 17 Ishikawa, 18 Fukui, 19 Yamanashi, 20 Nagano, 21 Gifu, 22 Shizuoka, 23 Aichi, 24 Mie, 25 Shiga, 26 Kyoto, 27 Osaka, 28 Hyogo, 29 Nara, 30 Wakayama, 31 Tottori, 32 Shimane, 33 Okayama, 34 Hiroshima, 35 Yamaguchi, 36 Tokushima, 37 Kagawa, 38 Ehime, 39 Kochi, 40 Fukuoka, 41 Saga, 42 Nagasaki, 43 Kumamoto, 44 Oita, 45 Miyazaki, 46 Kagoshima, 47 Okinawa. Population statistics for Japanese adults aged 20–79 were obtained from the Ministry of Internal Affairs and Communications, Basic Resident Registration Data 2020 (https://www.e-stat.go.jp/statistics/00200524 for sex and age distributions; https://www.e-stat.go.jp/dbview?sid=0003448233 for prefecture-level distribution; accessed 2025/11/26).

**Table S1. Key predictor variables used in the current study**

| Domains | Features |
| --- | --- |
| Demographic | sex, age, marital status, educational attainment, employment status, and health insurance coverage |
| Health-related | body mass index (BMI), alcohol use and alcohol dependence, tobacco use, use of sleeping pills, self-rated health, health literacy, quality of life (QOL), history of common physical illnesses, current physical symptoms, and history of psychiatric disorders, etc. |
| Psychological | personality traits, loneliness, fatigue, subjective happiness, and experiences of interpersonal victimization or violence, etc. |
| Financial | household income, changes in income, perceived financial uncertainty, and the presence of loans or other debt, etc. |
| Family- and household-related | bereavement, caregiving responsibilities, housing type, presence of children, parenting practices and activities, child abuse behaviors, and marital conflict, etc. |
| Lifestyle | time allocation for daily activities (e.g., screen time, walking, sedentary behaviors, sleep), regularity of daily routines, breakfast habits, nutritional patterns, and changes in behaviors such as exercise, sleep schedule, or toothbrushing, etc. |
| Work-related | industry of employment, workplace size, job type, weekly working hours, work presenteeism, job demands and job control, night-shift burden, workplace cohesion, general work environment, work engagement, workplace harassment, job stability, supervisor and coworker support, and remote work arrangements, etc. |
| Social | frequency of outings, interactions with neighbors, in-person visits with family, and levels of trust in one’s community and in government institutions, etc. |
| COVID-19-related | infection history, symptomatic experiences, work disruptions associated with the pandemic (e.g., work loss, leave, job loss, job change), preventive behaviors, pandemic-related loneliness, and fear or anxiety regarding COVID-19, etc. |


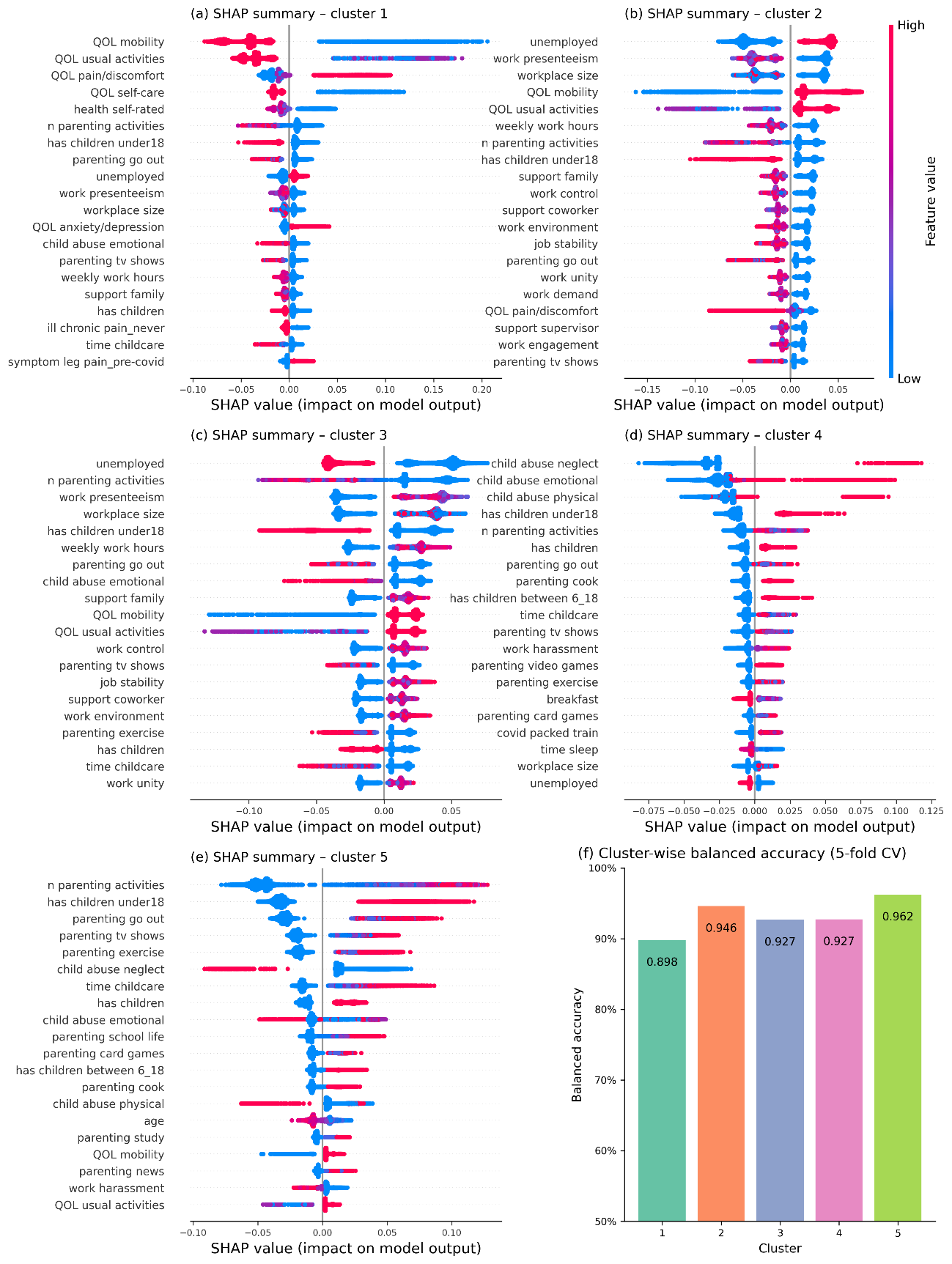


**Figure S2. SHAP-based characterization of cluster-defining features and cluster classification performance.** (a–e) SHAP (SHapley Additive exPlanations) summary (beeswarm) plots for each cluster, showing the distribution of SHAP values for the top features used by the Random Forest classifier when discriminating that cluster from the others. Each point represents an individual participant, positioned horizontally by the SHAP value (feature contribution to the prediction for that person) and colored according to the feature value. Features are ranked vertically based on their overall impact, with those at the top contributing most strongly. (f) Balanced accuracy of the Random Forest classifier in predicting cluster membership, estimated using stratified 5-fold cross-validation for each cluster. Bars represent per-cluster balanced accuracy, colored according to the cluster palette, with exact values displayed inside each bar.


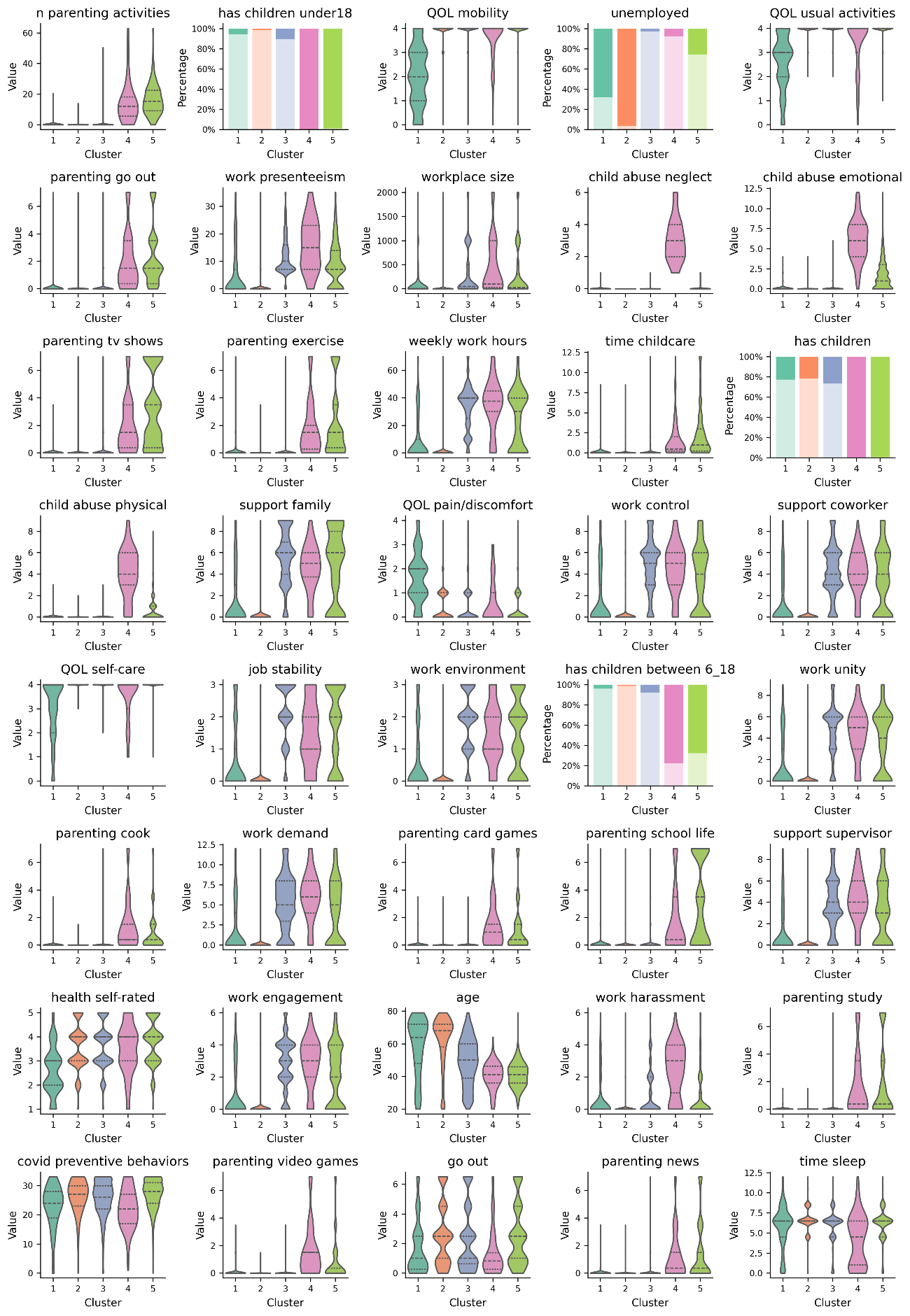


**Figure S3. Distributions of SHAP-identified top 40 globally important features across clusters.** For continuous and ordinal variables, violin plots show the distribution of feature values by cluster, including median and interquartile ranges. For binary and one-hot–encoded variables, stacked bar charts show the proportion of participants with values 0 (translucent segment) and 1 (solid segment) in each cluster, with bar color indicating cluster ID.


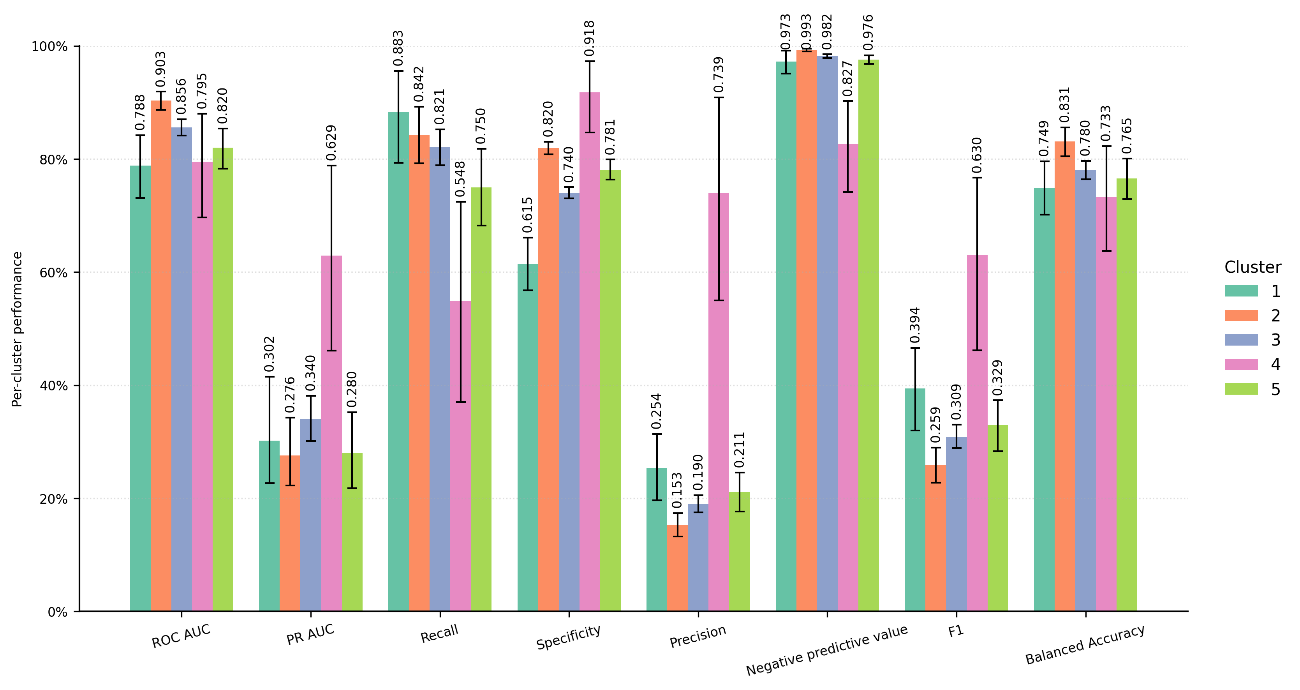


**Figure S4. Cluster-wise performance of Random Forest models predicting incident depressive and anxiety disorders at follow-up.** For each cluster, out-of-fold cross validated performance is evaluated across eight metrics: area under the receiver operating characteristic curve (ROC AUC), area under the precision–recall curve (PR AUC), recall (sensitivity), specificity, precision (positive predictive value), negative predictive value, F1 score, and balanced accuracy. Error bars indicate 95% bootstrap confidence intervals. Bars are color-coded by cluster, and exact metric values are displayed above each bar.


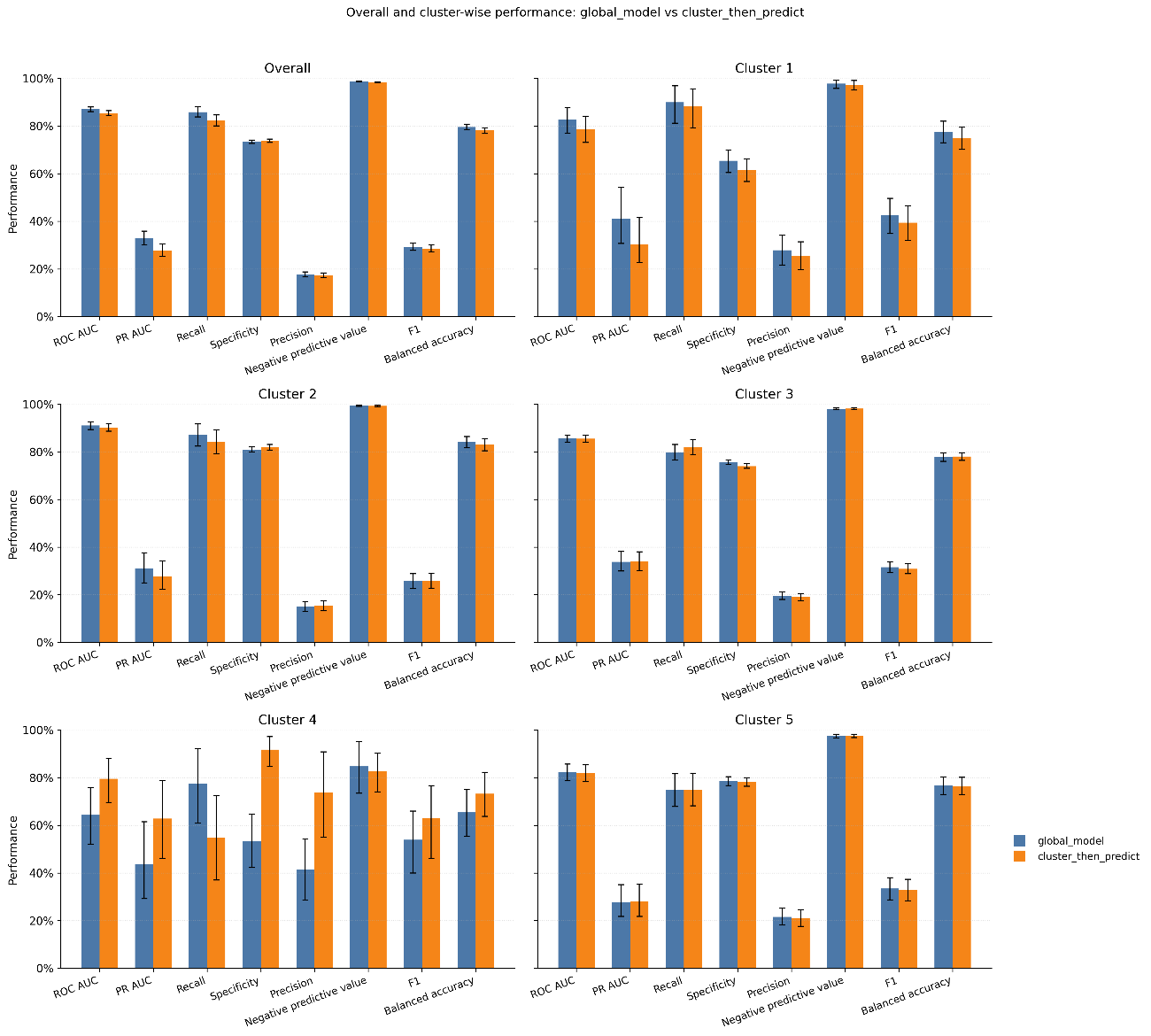


**Figure S5. Comparison of predictive performance between the global model and the cluster-then-predict framework.** Performance of the global Random Forest model (“global_model”) and the cluster-specific modeling framework (“cluster_then_predict”) is compared across all eight metrics. (a) Overall performance based on all participants. (b–f) Cluster-wise performance for clusters 1–5. In each panel, bars indicate point estimates and error bars indicate 95% bootstrap confidence intervals derived from out-of-fold predictions. Blue bars represent the global model and orange bars represent the cluster-then-predict framework.


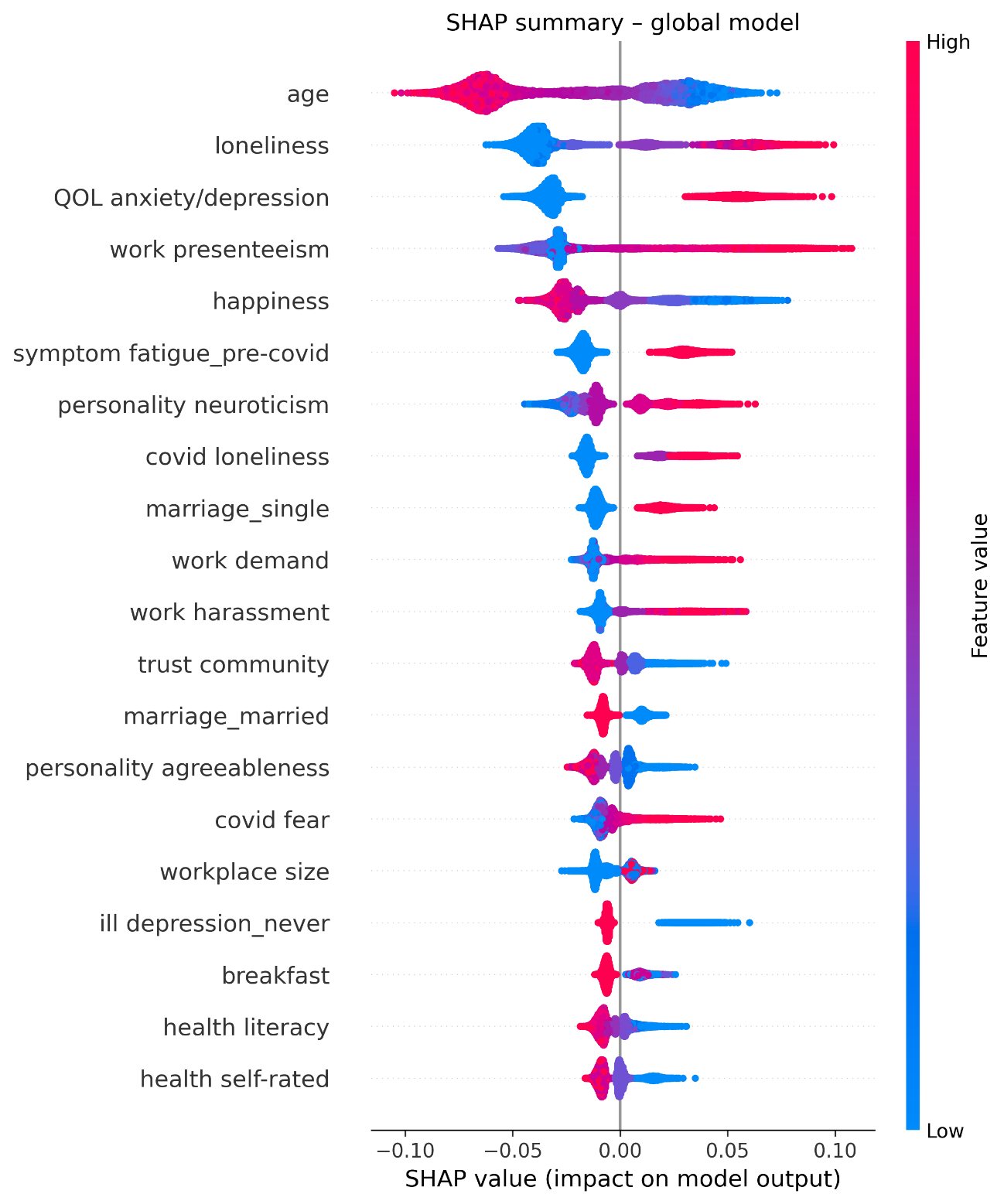


**Figure S6. SHAP feature importance for the global Random Forest model predicting incident depressive and anxiety disorders at follow-up.** Beeswarm plot showing SHAP (SHapley Additive exPlanations) value distributions for the top 20 features contributing to the global Random Forest model. Each point represents an individual participant, and its horizontal position indicates the feature’s marginal contribution to higher (positive SHAP) or lower (negative SHAP) predicted risk. Color indicates feature values (high in red, low in blue). Features are ranked vertically by their overall impact, with those at the top contributing most strongly.

**
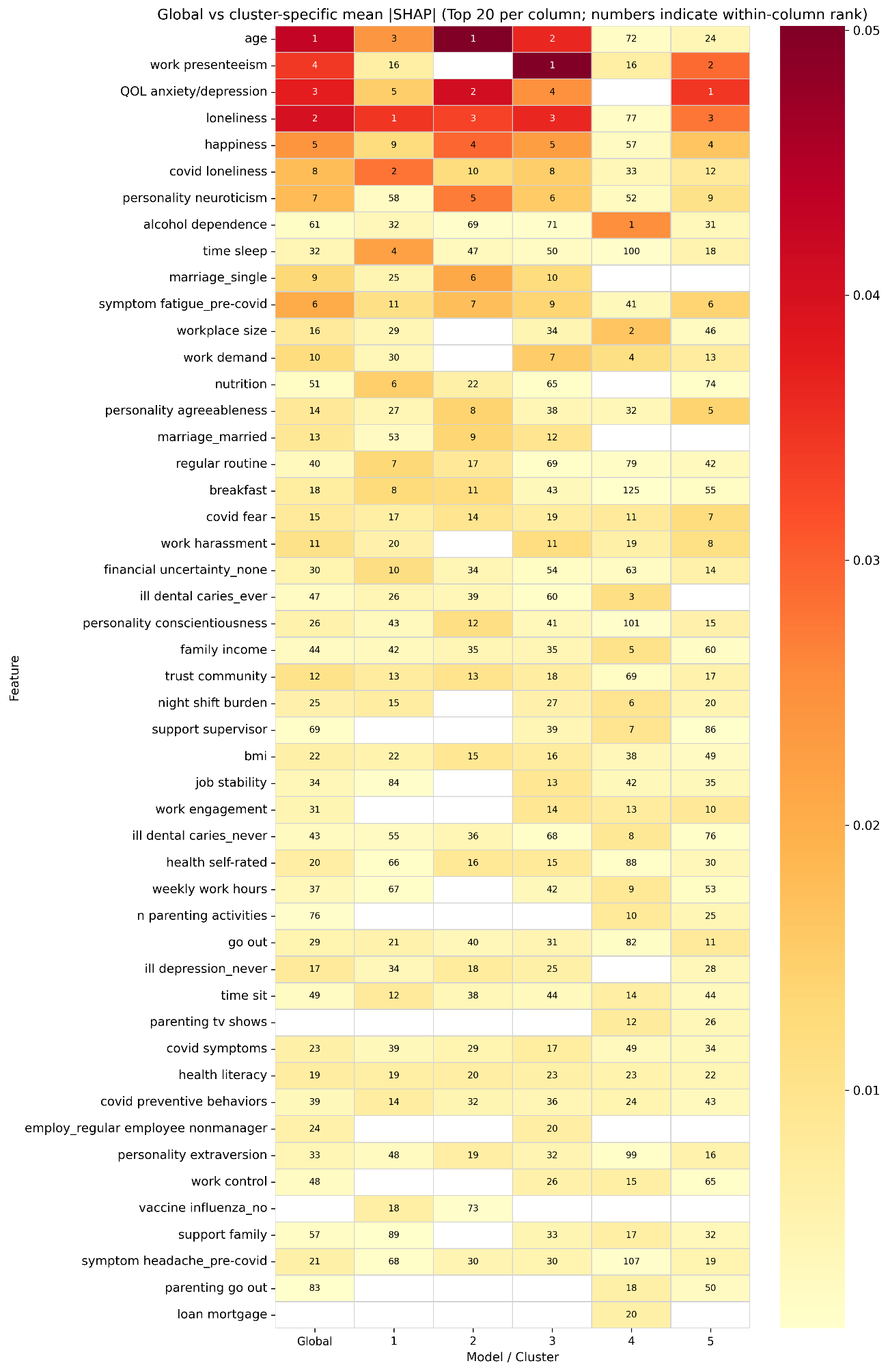
**

**Figure S7. Comparison of SHAP feature importance between the global model and cluster-specific models for predicting incident depressive and anxiety disorders at follow-up.** Heatmap displays the union of the top 20 features from the global Random Forest model and each cluster-specific Random Forest model. Rows are ordered by the maximum mean absolute SHAP value observed across all columns. Columns correspond to the global model and clusters 1–5, and cell color indicates the mean absolute SHAP value for that feature within the corresponding model (warmer colors = greater importance). White cells indicate near-zero importance (mean |SHAP| below 0.001). Numbers within colored cells denote the within-model rank of that feature’s mean |SHAP| (1 = most important).

Supplementary methods

The raw data were first cleaned according to the predefined exclusion criteria established by the Japan COVID-19 and Society Internet Survey (JACSIS; Okubo et al., 2021). Participants were excluded if they failed one attention-check item or either of the two straight-lining checks. The attention-check item instructed respondents to “choose the second option from the bottom of the following list”; those who did not respond correctly were removed.

Straight-lining was assessed in two domains: substance use and comorbidities. For substance use, respondents who selected the same extreme response options (“almost every day” or “occasionally”) across all nine queried substances (e.g., alcohol, sleeping pills, prescribed narcotics for cancer pain, cannabis, methamphetamine) were excluded. For comorbidities, respondents who uniformly indicated either “currently have this condition and receiving treatment” or “currently have this condition but not receiving treatment” for all 16 medical conditions (e.g., hypertension, diabetes, asthma, myocardial infarction, stroke, cancer) were removed.

In addition to the above JACSIS criteria, we applied several additional quality-control procedures. Respondents were excluded if they reported implausible household compositions (i.e., household size ≥ 15; ≥ 6 children aged ≥ 6 years; ≥ 6 children aged < 6 years; household size smaller than the sum of its composition), unknown educational attainment, mean daily sleep time of zero hours, or extreme BMI values (more than two interquartile ranges above the 75th percentile or below the 25th percentile).

The data analyses were implemented in Python 3.11.7 using NumPy 1.26.4, Pandas 2.3.3, SciPy 1.11.4, SciKit-learn 1.4.2, umap-learn 0.5.6, SHAP 0.48.0, Matplotlib 3.8.4, Seaborn 0.13.2.

References

Okubo, R., Yoshioka, T., Nakaya, T., Hanibuchi, T., Okano, H., Ikezawa, S., ... & Tabuchi, T. (2021). Urbanization level and neighborhood deprivation, not COVID-19 case numbers by residence area, are associated with severe psychological distress and new-onset suicidal ideation during the COVID-19 pandemic. Journal of affective disorders, 287, 89-95.
